# Supplementary material for: A cytoplasmic chemoreceptor and reactive oxygen species mediate bacterial chemotaxis to copper
Source: J Biol Chem. 2023 Sep 1;299(10):105207. doi: 10.1016/j.jbc.2023.105207 (PMC10579534; doi:10.1016/j.jbc.2023.105207)
Supplement: Supporting Information Figures [file mmc1.pdf]

Figure S1

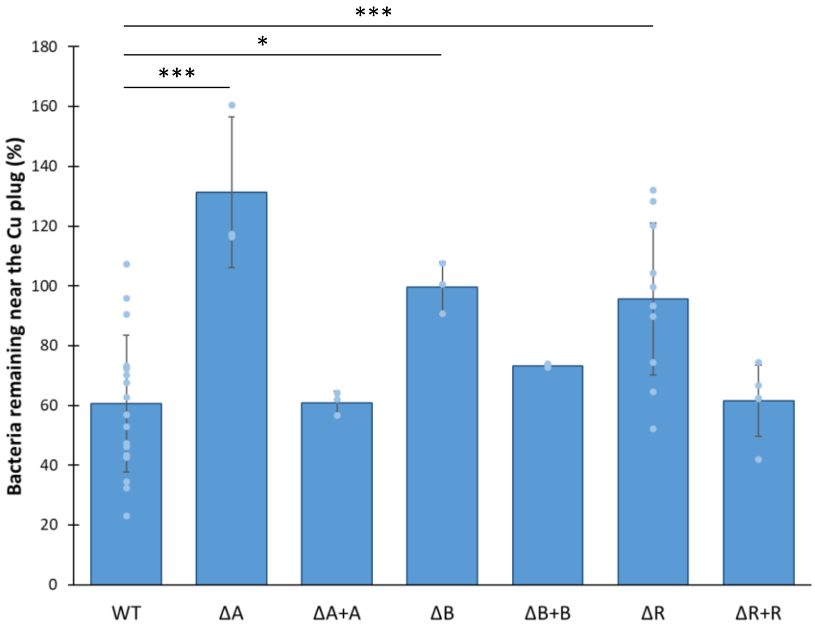

Figure S1: McpA, McpB and McpR are potentially involved in Cu chemotaxis. Percentage of WT, Δ*mcpA*, Δ*mcpA* overexpressing McpA, Δ*mcpB*, Δ*mcpB* overexpressing McpB, Δ*mcpR* and Δ*mcpR* overexpressing McpR SW cells remaining in the vicinity of the Cu plug after 25 min. Mean +/- s.d., at least 3 biological replicates. *p-values* were calculated using an ANOVA (* $p < 0.05$, ** $p < 0.01$, *** $p < 0.001$).

Figure S2

A.

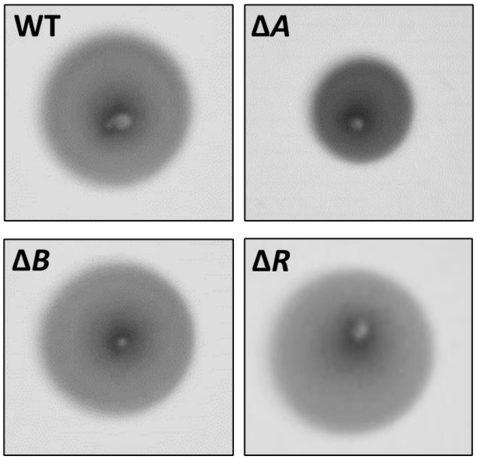

B.

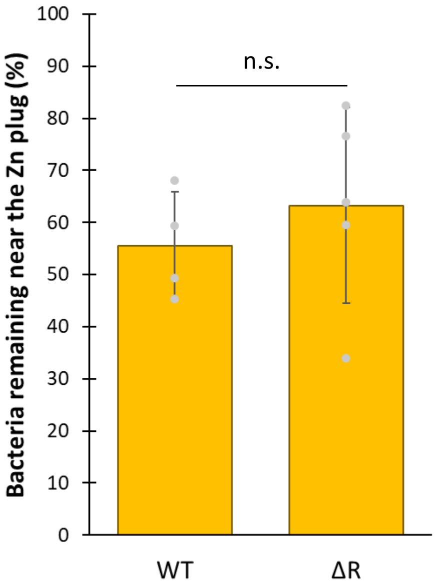

Figure S2: The chemotactic machinery is functional in the Δ*mcpR* mutant. A. Chemotaxis-driven swarming pattern of the WT, Δ*mcpA*, Δ*mcpB* and Δ*mcpR* strains in a Cu ions-free medium. **B.** Percentage of WT and Δ*mcpR* SW cells remaining in the vicinity of the Zn plug after 25 min. Mean +/- s.d., at least 3 biological replicates. *p-values* were calculated using a *t-test* (* $p < 0.05$, ** $p < 0.01$, *** $p < 0.001$).

Figure S3

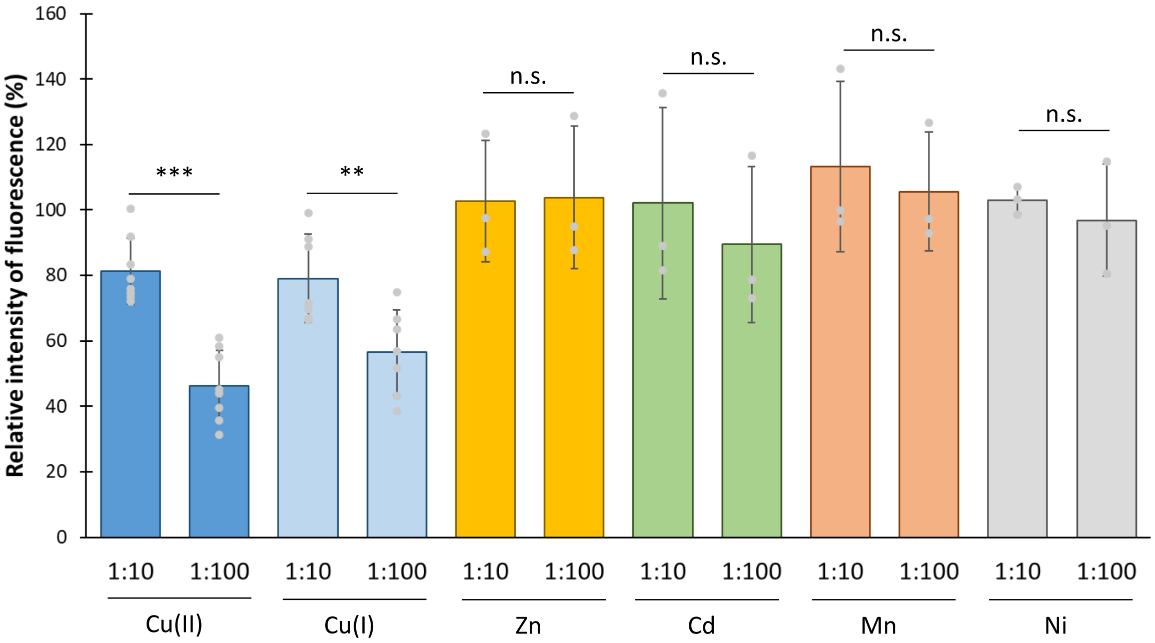

Figure S3: Zn, Cd, Mn and Ni do not directly bind to McpR. Relative intrinsic fluorescence of purified McpR (3.58 µM) incubated with Cu(II), Cu(I), $ZnSO_4$, $CdSO_4$, $MnSO_4$ and $NiSO_4$ at 1:10 and 1:100 ratios. Mean +/- s.d., at least 3 biological replicates. *p-values* were calculated using a t-test and were not significant (* $p < 0.05$, ** $p < 0.01$, *** $p < 0.001$).

Figure S4

A.

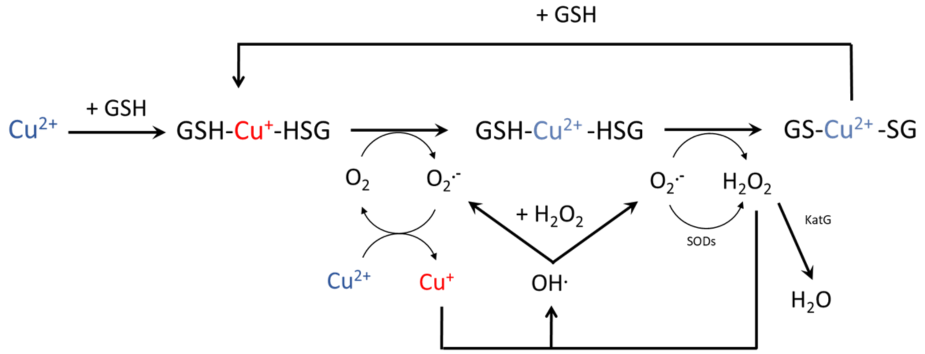

B.

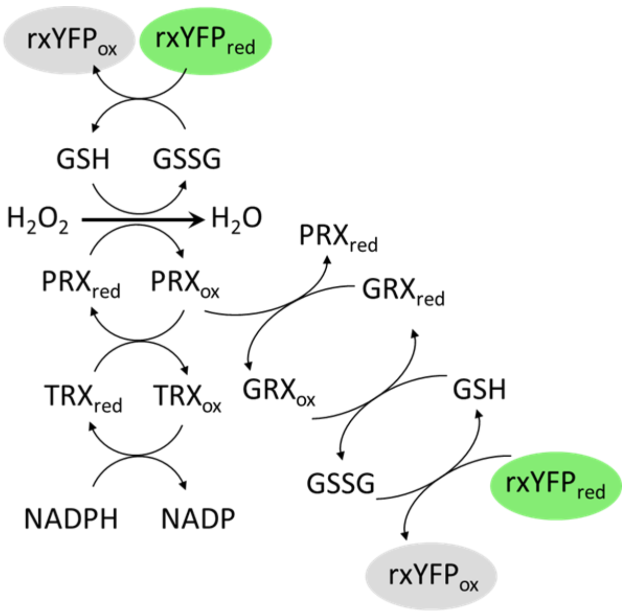

Figure S4: Potential pathways leading to ROS production by Cu. A. *In vitro* generation of Cu-induced ROS by Fenton-like reaction and Haber-Weiss cycles. The Cu-GSH complexes may also trigger ROS production (11). The SODs and KatG are involved in O_2**•-** and H_2O_2 detoxification, respectively **B.** The rxYFP biosensor is in equilibrium with the GSH pool. GSH is involved in different ways in ROS buffering. As a result, ROS production triggers an increase of the GS-SG pool, which will be partially reduced to GSH by using rxYFP as an electron donor. The resulting oxidized rxYFP will lose its fluorescence properties. GSH (glutathione), PRX (Peroxiredoxins), TRX (thioredoxin) and GRX (glutaredoxin).

Figure S5

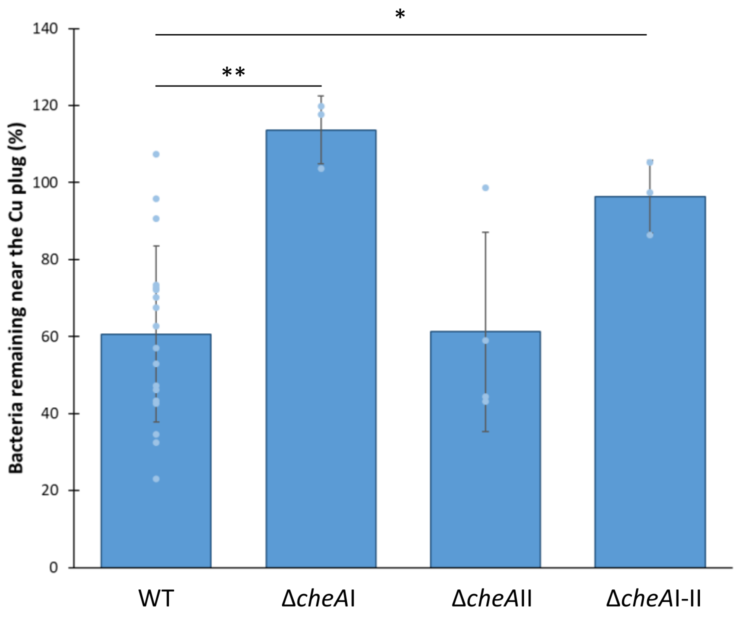

Figure S5: CheAI is the main histidine kinase involved in Cu chemotaxis. Percentage of WT, Δ*cheAI*, Δ*cheAII* and Δ*cheAI-II* SW cells remaining in the vicinity of the Cu plug after 25 min. Mean +/- s.d., at least 3 biological replicates. *p-values* were calculated using an ANOVA (* $p < 0.05$, ** $p < 0.01$, *** $p < 0.001$).

Figure S6

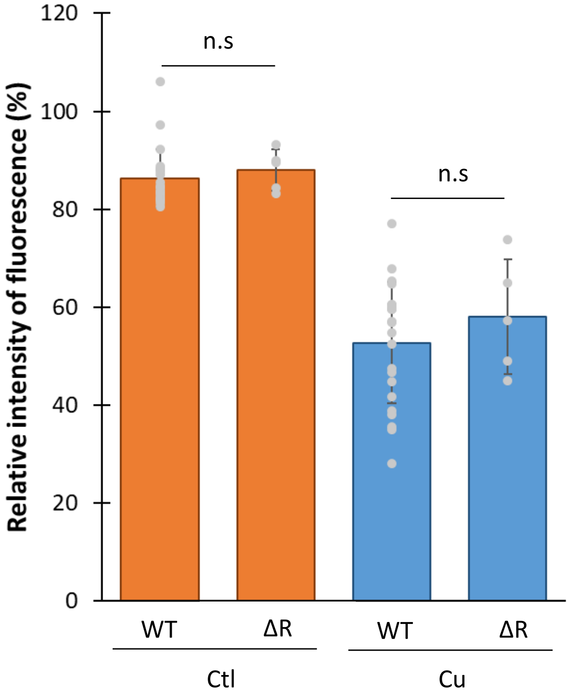

Figure S6: The Cu-induced oxidative stress is not affected in the Δ*mcpR* mutant. Relative intensity of fluorescence of the WT and Δ*mcpR* SW cells expressing the rxYFP biosensor and exposed to 175 µM Cu for 20 min. Mean +/- s.d., at least 3 biological replicates. *p- values* were calculated using a *t-test* (* $p < 0.05$, ** $p < 0.01$, *** $p < 0.001$).

Figure S7

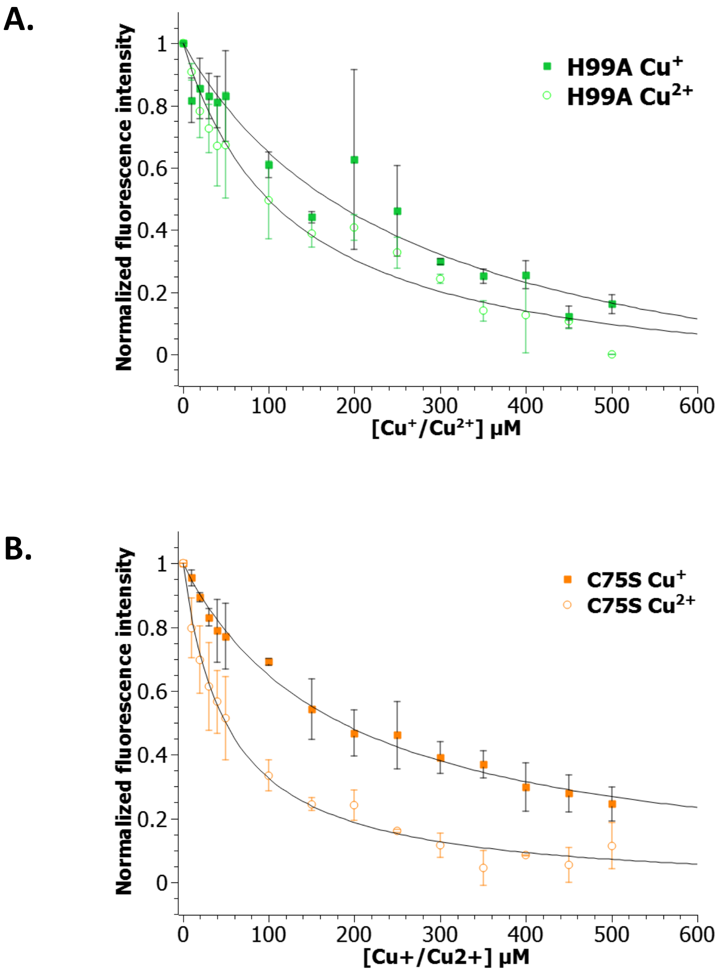

Figure S7: H99A (A) and C75S (B) McpR titration by Cu(I) (full squares) or Cu(II) (circles) monitored by protein intrinsic fluorescence intensity quenching, fitted to a single-site hyperbolic model by non-linear regression analysis (solid line). The fluorescence intensity changes are normalized to the maximum fluorescence intensity change.

Figure S8

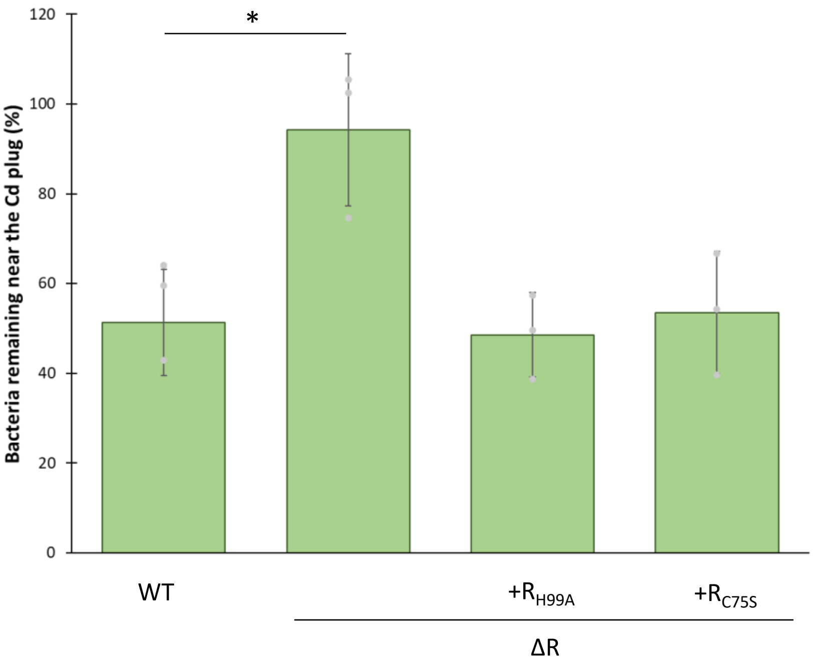

Figure S8: The McpR$_{C75S}$ and McpR$_{H99A}$ mutants are still functional. Percentage of WT, Δ*mcpR* and Δ*mcpR* overexpressing McpR, McpR$_{H99A}$ or McpR$_{C75S}$ SW cells remaining in the vicinity of the Cd plug after 25 min. Mean +/- s.d., at least 3 biological replicates. *p-values* were calculated using an ANOVA (* $p < 0.05$, ** $p < 0.01$, *** $p < 0.001$).
