# Supplementary material for: A cytoplasmic chemoreceptor and reactive oxygen species mediate bacterial chemotaxis to copper
Source: J Biol Chem. 2023 Sep 1;299(10):105207. doi: 10.1016/j.jbc.2023.105207 (PMC10579534; doi:10.1016/j.jbc.2023.105207)
Supplement: Supporting Information Tables [file mmc2.pdf]

Table S1: *pvalues.* One-way ANOVA and t-test were performed using GraphPad Prism.

**Figure 1A (ANOVA)**	**pvalue**	**summary**
WT vs. ΔA	<0.0001	****
WT vs. ΔB	0.0338	*
WT vs. ΔC	0.9993	ns
WT vs. ΔD	0.9992	ns
WT vs. ΔE	>0.9999	ns
WT vs. ΔF	0.6094	ns
WT vs. ΔG	0.9991	ns
WT vs. ΔH	0.8952	ns
WT vs. ΔI	0.9689	ns
WT vs. ΔJ	0.999	ns
WT vs. ΔK	0.9991	ns
WT vs. ΔL	>0.9999	ns
WT vs. ΔM	0.9796	ns
WT vs. ΔN	0.9993	ns
WT vs. ΔO	0.9996	ns
WT vs. ΔP	0.9996	ns
WT vs. ΔQ	0.9988	ns
WT vs. ΔR	0.0001	***
WT vs. ΔS	0.9995	ns
**Figure 1D (ANOVA)**	**pvalue**	**summary**
50 μM Cu vs. 100 μM Cu	0.0128	*
50 μM Cu vs. 175 μM Cu	0.0011	**
100 μM Cu vs. 175 μM Cu	0.2469	ns
**Figure 2A (ANOVA)**	**pvalue**	**summary**
Ctl vs. Cu	<0.0001	****
Ctl vs. H2O2	0.0007	***
**Figure 2B (ANOVA)**	**pvalue**	**summary**
Ctl vs. Cu	<0.0001	****
Ctl vs. H2O2	<0.0001	****
**Figure 2C (ANOVA)**	**pvalue**	**summary**
Ctl vs. 20 μM	<0.0001	****
Ctl vs. 175 μM	<0.0001	****
Ctl vs. 300 μM	0.0004	***
Ctl vs. 1.16 mM	<0.0001	****
**Figure 2D (ANOVA)**	**pvalue**	**summary**
WT vs. sodB+	0.0005	***
WT vs. katG+	0.001	***
**Figure 2F (ANOVA)**	**pvalue**	**summary**
WT vs. cheAI	0.0007	***
WT vs. sodB+	0.0156	*
WT vs. katG+	0.0045	**
**Figure 3C (ANOVA)**	**pvalue**	**summary**
WT vs. ΔR	0.0001	***
WT vs. ΔR+R	>0.9999	ns
WT vs. H99A	0.2356	ns
WT vs. C75S	0.2503	ns
ΔR+R vs. C75S	0.0056	**
ΔR+R vs. H99A	0.0053	**

**Figure S1 (ANOVA)**	**pvalue**	**summary**
WT vs. ΔA	<0.0001	****
WT vs. ΔA+A	>0.9999	ns
WT vs. ΔB	0.0338	*
WT vs. ΔB+B	>0.9999	ns
WT vs. ΔR	0.0001	***
WT vs. ΔR+R	0.9954	ns
**Figure S2 (t-test)**	**pvalue**	**summary**
WT vs. ΔR	0.4878	ns
**Figure S3 (t-test)**	**pvalue**	**summary**
Cu(II) 1:10 vs. Cu(II) 1:100	1.0372E-05	***
Cu(I) 1:10 vs. Cu(I) 1:100	0.00784501	**
Zn 1:10 vs. Zn 1:100	0.9479843	ns
Cd 1:10 vs. Cd 1:100	0.59277248	ns
Mn 1:10 vs. Mn 1:100	0.70207278	ns
Ni 1:10 vs. Ni 1:100	0.58457574	ns
**Figure S5 (ANOVA)**	**pvalue**	**summary**
WT vs. Δ*cheAI*	0.0019	**
WT vs. Δ*cheAII*	>0.9999	ns
WT vs. Δ*cheAI-II*	0.0406	*
**Figure S6 (t-test)**	**pvalue**	**summary**
WT Ctl vs. R Ctl	0.9651	ns
WT Cu vs. R Cu	0.6783	ns
**Figure S8 (ANOVA)**	**pvalue**	**summary**
WT vs. ΔR	0.0171	*
WT vs. H99A	0.8499	ns
WT vs. C75S	0.9949	ns

Table S2: Strains, primers and plasmids (1/4)

Strains	Relevant genotype or description	References
C. crescentus		
WT	CB15N (or NA1000), synchronizable variant strain of CB15	(Evinger and Agabian, 1977)
WTrxYFP	WT strain carrying the *rxYFP* gene on the pJS14 under the control of the lac promoter; CmR	This study
ΔcheAI	Knockout strain for *cheAI* gene (CCNA_00442)	This study
ΔA	Knockout strain for *mcpA* gene (CCNA_00439)	This study
ΔA+A	Knockout strain for *mcpA* gene carrying a copy of *mcpA* on the pMR10 under the control of the lac promoter; KanR	This study
ΔB	Knockout strain for *mcpB* gene (CCNA_00437)	This study
ΔB+B	Knockout strain for *mcpB* gene carrying a copy of *mcpB* on the pMR10 under the control of the lac promoter; KanR	This study
ΔC	Knockout strain for *mcpC* gene (CCNA_00348)	This study
ΔD	Knockout strain for *mcpD* gene (CCNA_01727)	This study
ΔE	Knockout strain for *mcpE* gene (CCNA_02364)	This study
ΔF	Knockout strain for *mcpF* gene (CCNA_02773)	This study
ΔG	Knockout strain for *mcpG* gene (CCNA_00626)	This study
ΔH	Knockout strain for *mcpH* gene (CCNA_03459)	This study
ΔI	Knockout strain for *mcpI* gene (CCNA_02940)	This study
ΔJ	Knockout strain for *mcpJ* gene (CCNA_03247)	This study
ΔK	Knockout strain for *mcpK* gene (CCNA_00629)	This study
ΔL	Knockout strain for *mcpL* gene (CCNA_03468)	This study
ΔM	Knockout strain for *mcpM* gene (CCNA_02402)	This study
ΔN	Knockout strain for *mcpN* gene (CCNA_00538)	This study
ΔO	Knockout strain for *mcpO* gene (CCNA_02935)	This study
ΔP	Knockout strain for *mcpP* gene (CCNA_01465)	This study
ΔQ	Knockout strain for *mcpQ* gene (CCNA_00064)	This study
ΔR	Knockout strain for *mcpR* gene (CCNA_02901)	This study
ΔRrxYFP	Knockout strain for *mcpR* gene carrying the *rxYFP* gene on the pJS14 under the control of the lac promoter; CmR	This study
ΔR+R	Knockout strain for *mcpR* gene carrying a copy of *mcpR* on the pMR10 under the control of the lac promoter; KanR	This study
ΔR+RH99A	Knockout strain for *mcpR* gene carrying a copy of *mcpR* with the mutation H99A on the pMR10 under the control of the lac promoter; KanR	This study
ΔR+RC75S	Knockout strain for *mcpR* gene carrying a copy of *mcpR* with the mutation C75S on the pMR10 under the control of the lac promoter; KanR	This study
ΔS	Knockout strain for *mcpS* gene (CCNA_00160)	This study
sodB+	WT strain carrying a copy of *sodB (CCNA_01855)* on the pMR10 under the control of the lac promoter; KanR	This study
katG+	WT strain carrying a copy of *katG (CCNA_03138)* on the pMR10 under the control of the lac promoter; KanR	This study
E. coli		
S17-1	RP4-2, Tc::Mu, KM-Tn7, for plasmid mobilization	
BL21	BL21 *E. coli* strain used for overexpression of proteins	
**Plasmids**	**Relevant genotype or description**	
pNPTS138	mobRP4+ ori-R6K sacB; integrative vector in *C. crescentus* for in-frame deletions; KanR	
pMR10	DH10B strain carrying the pMR10 low copy number replicative cloning vector in *E. coli* and *C. crescentus*; KanR	
pJS14	DH10B strain carrying the pJS14 medium copy number replicative cloning vector in *E. coli* and *C. crescentus*; CmR	
pET28a	BL21 strain carrying the pET28a high copy number replicative cloning vector in *E. coli*; KanR	

Table S2: Strains, primers and plasmids (2/4)

Target	Sequence
Gblock used for the KO of *cheAI*	TGGCTTCATCGAGGCGGTGCGCGTCGACGACGACTACCGCGCGATCCCGATCCTGGTGCTGACCACCGAAAGCGACCCTGCCAAGAAGCAG<br>CGGGCTCGCGAGGCGGGCGCCACGGGCTGGATCGTCAAGCCGTTCAACCCGGAAAAGCTCGTCGACGCCATCCGCCGCGTCGCCGCCTGAT<br>CCCGGTCACGGACCTCAGTACCCCGTTATTGAATACCCGTTGCTAGACGCGAATTAGGACTTACGGGACTGACGCTGGGCTATCGCGAGGAA<br>AGTCCGCATCCGACGGATGGCGTGGTGCTGCTGGTCGAAGGCGAAGACGGCTCGCGGGCCGCCCTGGTCGCCGACGCCATCCATGGCCAG<br>CGTCAGGTGGTCATCAAGTCTCTGGAGCAGAACTATCAGCAGGTCGAAGGCGTCGCCGCCGCGACGATCCTCGGTGACGGCCGTGTGGCTC<br>TGATCCTCGACGTCGACGCCACGATCAACCTCCGCCGTCGCGAAG
Gblock used for the KO of *mcpA*	ACTGGACGCAGGAGCAAATTCGCAGGTGCAAGTTAAGATATTCTGAGTCGCACGGTAAGATTGCGGGTTTACTTTCAGGTAACCACGATCTG<br>CCGCTTTGGTGACTCTCATTGTGCGGCGAGGGTTTCGCAGATTCCATGCGGCGGAGCCTGCGACCAAACGACCACATGCTCCAAAATGGGAC<br>GAAAATCTCTGACTTCCTTTAACCAAGTCAGAGTCTGTTAGGAATCTATCGGGATTATCACCCTTCTGATGGCCCGCTTCCAGGTGGGCTCCG<br>GTTCGTCGTCCTACGCCCGTCCGGCCGTCGCCGACGCGGGTCATCACGCCCCCGCGCGAAATCCGGTGGCCGAGCAACAGGCCCGTCTGAA<br>CACCTTCGCCCGTCCGGGTCGGAGCAGCGGTTCGGCGGCCCTCGCCCAGGCCCCCGCCTCGGATGGTTGGGAGGAATTCtaaTGGTTGCCGC<br>GATCTCGCTGCCCGAAAACCTGGATCTCAAGACCGCAGCC
Gblock used for the KO of *mcpB*	CCAGGTCCAGCAGCTGATCATTGCGCGTGAAACGCTGAAGCGCGGCGGGTGAGTGCGACACCCTGTCGCCCCCTCCGCCCGTCGGCCGTAT<br>GCAATCGATAAACCATCCCCTCGTTCCCACGATATTGACCGTTCAGTCTGTGCAAACGGTTGCAACCTGAGCGGGGGTGCGGCGAGGCGCCG<br>CACTCGCTGCGAGAATATTTAAGCGCTCATTAGGGATAAGGGTCCGAGGATTTCCCCAAGCGAAACACGCGCGGACCCTCGAGCGCCCTGG<br>GTTCAGGCATGTTCGCTTAGACCGTACGAACGCGCTCAGCAGATTTGGTTCGGGAGCTTCTCGACAGCGGGGCCGTGTCGTCTTGGCGCGGT<br>CTCTCGTCGTTCCGGCGCCAACATCGCCCCAGACAGCAGAACGCCCCCGCCGACCAGGTCGACGAGGGCGTTTTGTGTTCCGATCGGAAAGA<br>CGCTTACGCGCCGCCCGGGAAGCGGAACGGAACCTTCACGGT
Gblock used for the KO of *mcpC*	CGACCACGCAACGTTACACCCAGGTGGACGCCGCCGGCCTGTTGGCCGCCTATCAGGCCGCCCACCCTAAGGGATGACCCCGAGCAAGCAC<br>GCCGGGAGGTTCATCGCGCCCGTCCGTCGCGATGCGACACACTGTCGCGAAGGGCGACCGCCGAAACGCCATTAACCGCGATTTTACCGCA<br>TCAGGGCGTTGTGGGGGCTTGAGAAATTTCCCTCTGCGTCCCGCGTCTGATCGCTGACGAGGCCCCCTACCGGTTTAGTAAGCGTAAGCCCC<br>GCCGCCCCCCTCTGGCGGGGTTCCCCCCCTCGAAAAACAGACTGGCCCCGCCTCCTCAAGAGGCGGGGCCTTTTTCTTGGCTGTATTCGCGTT<br>GAGCGTGGGCGCTCACGCGGATTGGAAATCCTCGTCCTTCGACAAGCTCAGGATGAGGATTTTGTGCGAACCGAGGCCAACAGTCGTCCTC<br>ACCCTGAGCTTGTCGAAGGGCGAGGGCGGCCGCCACGACGCA
Gblock used for the KO of *mcpD*	TCGCGCCGTTCATGGTCGATCTGCTGCGCACTGATCGCGAGGCGGCCAAGGGTGTGGTTCGCTTCAAGGAACGCGGCCAGGAATCAGCGTC<br>GATCGACGCCTGACCGATTCGGCGGCGCCGCCTTCATGGACGCAAGGGTTGTCTGACGCTGTCAGACTTCCACTCTGGATGGCGATTCTAGA<br>ACCGCGTTCGGCGATAGGTCGCCCCAAACGGGAGCATTAGCGGCTCGTTAACCATGCACGGCCAGTTTCCCGTGGGGGGACGAACCCGCAG<br>TAACGGGTTCCAGATGGCCGCCGCTGACAAAGCGGCGGCCATTTTCGGCTCTGCTTAGCGCCCTTTAGGGCGACCGGGCCCGGTGTTCCCAC<br>GCACTGTGCGCGAACCCGCAGCCGGACGGCGCGGCGCGGCGGCCTTGCCGCCGGCCGTCTTGGTCCCGACCTTGTAGCTGGGCGTCGTGGC<br>GACCTTCTTCTGGGCGCGCGGTCCCTTGGCCTTCACGGACTTG
Gblock used for the KO of *mcpE*	CGAGGGGACCGATTCGGCCACGCCCCTGGCCTGGAGCCACGCGGAGTACGTCAAGCTGCTGCGATCGGTCAGCGACGGGCAGGTCTGGGA<br>TCACTATGCGCCCGTAAAGGCGCGGTACGCGCGCTAGAGGGGCCAAAAAGTGCGCGACAATGTCGCGGTCGCGCATTCCTGAAACGTGGTT<br>AACGGGGCTTTAGGCATTAGGCCGTGAACTTGAGCGTAATCGCCTAGCTCACGGCCGGAGGCCTTTGTTCGCTCCGACGCGCGAGAGCTTCC<br>GGCAGCGCTACGTGCAGGGCGCCAACGCCCTGAAGGTCGCCCCCAGCTCCCGTCCCGGGGAATGGGAAGAGTTCtagGCCTGACCGCTCTCA<br>GCCGTGCGCAGGGCGAAACGGAGCTCTGACAGAGCGATACCGTTCTGGGCCTTTTCGGCTTCGCGAACGCTGACCAGGAGCTCATTCATGC<br>CATTGGCGACGGCCCAGTCCCAATGGGCGTGACCTGGGGTCGCC
Gblock used for the KO of *mcpF*	CCTGTTCTTCGGCCTCGATCACGCCCGCCAGAAGCTCGCTGCCTGGGCCGAAGACTACAACACACGCCGGCCGCACTCGTCGATCGGCTACC<br>AGACCCCCGCGGCCTATGCCGCGCACCTGAACGCAACAGGCCGACCCGCTGCGCAATGTGGGAGCTCCACGGCTCGGCCTGTTGCTACAAC<br>CGCGCCATCGGGCGTAACCAATGCCGAGACTCTGGTCCGCGCTGGATGAAAGATCAGAGGCAGGTCAGTCATGCTTTGGCCATGGAGGCTG<br>ACAACCTGACCGTACTGATGGGGCAGTTTCGGATCGGAGAAACTCCAGAAGCGCAGTCCAATCGCAGGGACTTAAGCAAAGGCGCCGGCG<br>TCCCGTCTAATTTGCGACCGATAGCGCAAAGCCACGCTCGTGCGGGATCCGCAGCCGCAAAGATTGATGGCTGGGACGAGTTCtaaCCCAGA<br>ACACAATGGTCTCTGCGCGCGCCAAAGGCGATCAAAAGGCCCGT
Gblock used for the KO of *mcpG*	TCACCGCGCTTCCGGAGCCAGAGTCTCCCTCCGAGGCCCGTCAACGGGAGCTCTGGCTCAACGGTCCCGCGCGTCCCGCCGAAGGACTCTCG<br>CAGGCCGACGCTGACCGTCTTTTCGAATAGCCATCGGCCCAGCGTGGCCGTGTCGGCTTGTTAACCGTTTCCGCCAGATAGTCGAACCTGGG<br>TTCAAGGTCAGCGATCatgTCGCCCCATTCATTCTCGCCGAAGGCGCTAACAGGTTCGATCAGCCGGCTACGGCGCGCGCTCGCCCCTGCAGC<br>GTTCACACGTCGCGCGGCGCCGCAGCCTAGGACGCCGAACGCACCATCCGCTCGAGGCGACGCAGCAGCACCCGCGCCTCGAACGGCTTAC<br>CGATAAAGTCGGCGGCCCAAGGCTCATCGACCGGCGAATATCCTCGGGCGAGGTCTGGGCGGTCAGCATCAAGGCGGGGATCTCCGCGA<br>ACGCGGGATCGCGACGCACCGCGTGGAGCAAGGCCAGGCCAT
Gblock used for the KO of *mcpH*	ACGCCCCCAATCCGAGCTGAGCGACAAAAGGCCACACTATTCGAAAGGTTatgTTTTACCCGAAATTCACCGAGACAAATCAGCGTTATATGG<br>ACTTGGGCGGTGTTCCGCCTCGCAAGACGCACGGATCGCGGCGAATGATCAAACTTCTGAACGGCTCCGCGGGCGACGCCAGCCAGAAGAT<br>CGACGCACTCGACAAGTCGCTCGCGATGATCGAGTTCGACGTCAAGGGAACGATCCTCGCGGCCAAGATCATCAACCGCCCCCCGCGCGCC<br>CCCCGCGCCGGCTGATGGGCCACATCAGATCGGGTGAAGAGAGCTGTCCTTGGCCGCCGACAAGGGGGCCGGCGGCGCGATCGGCGCTCG<br>AACCGTCTTCCGCGCCACATAGCGGCGCACACGCTTGCGGGTCTTCTTCAGCAGGCGCCAGCGGGCCTTGCGCAGGACCAGCTTCTCGGTGC<br>GCAGCATCTGTTGCCAGACCACCAGCAGCACCTCCGGCTCGCG
Gblock used for the KO of *mcpI*	GGCTTCGGAGCCTGTATCGTCatgAACGTCACCTTCTCCAACCTCGACGCCCAACGCCAGATCGGCGGCAAGCTGATCATCGCCCTCGCCTGG<br>CTGCTGATCCCGATCGTGCTGGGCGCGCGCCTGGCCGTGCAGGCGCCCATCCTGGGGCTCTCGATCGCCGCGGTGCTGGTCGCCACGACCCT<br>CAACACCGACGCCACCCAGCTGGCCGATCTGGTGCGCCAGTTCGACCTACCGCGAGGCCATATCGAGCAGTCCCAGCGGCGGGTCGCCtagG<br>ACGCGGCGCGGCGCCCTTTCGCGCCGCGCCGTTGCAAGCTCAAGCTTGGCCCCCTTGCGTTACCGGAACCGGGCGCCTATCCTCCCAGCCCC<br>CGGTTGTGCGCGCCCGCGACTGGAAGATCTTCCTCCCAAGGCGTCCGAGCTAAAATCCGCAATGGAAAAA
Gblock used for the KO of *mcpJ*	CCTCGGAATTCTTTTAGTCTTGCGACTTTGGACACCTAACGCCGACGCCTCTTAAGACCGTATAAATTACGACCAGTCTGTCTGGTGACACTAT<br>GTCGCATGGTTAGGAAGGCGCCACCGAAACGTCGTTAAGAGGCGAGCAATCACTCTTCTATAGGTCTGGGGGACGGCCGCGTGGCGCTAG<br>GTCCGTCGCATAGGCGATTTCGCCTAGAGGACGGATCAGCGCCGCGGCCGCGCTGAGGACCCGCCACCCCGGCGGCGGCGGCGCCGCCGC<br>CCCCTCCATCAATCCATCCATCCATAGCTATAGCGTCGAGCGCCCCCACCGGAGCGCTCGCGCCGGCACGCTTTCGCCTCGCGAGCCAGAGCT<br>CGCGCTGCAGATCATGGCGATTGTAACCACGACATAATGTCGCACCACAAAGTCGCTGCTGATAAGTTCGGGTTTACCTTGTTGGTATAGTA<br>GAAAAATCAACAAACACCCAAAAGTATATATAACTAAGAAA
Gblock used for the KO of *mcpK*	GCCTAGTAGGTTCAGCTCGACGCTAAAAGGCTTCAGTATTCCTCTAAACTCGCCACACTCAAAATGAGTATAGATCGCGCCTCTTAGAGTTAT<br>TCGGGGCCGGGGCTCGGGGCGGGTAACGCGCGGTTAATCGCGCGGATCAGAGATTGACGACCCATGCGATTTGCCCAGGCCGCCTCCATTC<br>CTCGGATGAAGGCGACGGGTCGGAGCGCGATGAAGGCGGGTTTTAAGACTCTGCGGCGTTGGGCCCGCGAGATCGCGCGCAACACCTCCG<br>AAGCCGCCAACGGCGCTCAAGACGTGTCGCGGAACATTTCCGACGTCTTGCATGGCGCCCAACAGACCGGCTCCGCCTCGCACCAGGTGGT<br>CAGCGCCTCGCAGGAGCTGGGGCGTCAAGCCGCAGCGCTGCGTGACCGCGTGGACACCTTCCTGAAGACGGTGCGGGCCGCCTAGCGCGC<br>TCCGGCGCGGTTAGGGCCTTCGGAGATAGCGATGGATGATCTGAT

Table S2: Strains, primers and plasmids (3/4)

Gblock used for the KO of *mcpL*	CAATCTGTCGCGGGTATGGTTACGGCCGCGTTTACCATCGGCCGAGCATCGTTGCAGCGCAGTTCAAGCTTGATCATTCAACCCCAAGCCCC<br>GGGTTCAGatgCTGCGCCTCAACGCCTTCCTGAAACTGCTGACCGTCGCGTTGATGGTCGGCACAATCCTTGCCATCGCCGCAGCGACGTTCG<br>GCCTTTTGCATCTGCGGGTCGGCGGACCGATCGCCAACCGTCAAGCCGCCGCCTTCGATCTCGTCAGCCCTCTAGCTTTGGATGGGAGCATCC<br>AAAGCGTTGGAAAGAGATCTAAATATCTGATTTTAAAGCCTTACATCCGCTCTGGCGCTTCGATGCCCAGCAGGTCCAACGCCAGCTCCAGCT<br>GGCGCAGGGTGGTCTCGGCCAGGGTCAGGCGCGAGGCGCGCAGCGTCTCGGTGTCAGCGCTCATGATCGGGCAGGCCGCGTAGAACTTCG<br>AGAACGACTGGGCCAGCTTGTAGGCGTGCTCGGCCACGA
Gblock used for the KO of *mcpM*	GTCGCAGCGGTTGACGCCGGTAAGGTCAAAGCGCAGGTCGCGACGGTCTTCGAGCGCCTCGGTGAGGCGAGGCCCGGCGTCAAACAGGCC<br>CCGCGCCGTCCAGTCCCCGGCGAGTACGGCGACCAGGCCTCCGTCCTGTTCGGTAAGGGTGAAGTCCGCCGGAAGCGCCATGAAGTCCTTG<br>GGGTCTGGTCCCGGTTATATCCAAGCCCACGCAAGGGCCTGACAAGCCCAAAACGCGCCAAAGCGCAGCCACGGCCCTCGGCCAAGTGAAC<br>GCCGCCGTGACCCAGCTCGACCAGGTGACCCAGCGCAACGCCGCCATGGTTTCGCAATCGACCGACGCCACCCACGCGCTCCGTGTCGAGG<br>CGGCCGATCTGTCGAACCGCGTCGGAGCTTTCCGCCTGGGGGCTCGGTCACAGCCGACCCATCAGGAACAGCCCGCCGAGAATCCCGTGCA<br>CGCCGCCCGCGCGCGCGTCGCCGCTTTCGCCCGTCCCGGGCGCTGA
Gblock used for the KO of *mcpN*	CGGCCGAGATCGCGCTTTCGGACGCCGAATAGGCGCCCGAAACAGTCGAAGACCCGGACGCCAAAAGTGTCCGGGGCGACGCCGAGCAAG<br>GTTTTCGCCAATTGATTGAGGAAAGGCCCGGGAGAGATCCCGGGCCTTTCTTTTGGGTTGAGTAGATCGCCCACAGATTGGGTAACCGGTG<br>GAACAACGTTTTCCTTGCGTTAAGAATCGACGCGCATCTTTCCTTAAGGGGAGTATGCAAACGATGTCAACAAGCGCCGAGACCGTCCGAAA<br>GAGCGTCACGGCGCCGGGAGGAATGTCACAATCACGCGCCGGTACCGGGTCAGGGCCGGCGCGCCCTTGTCGCTGATCGCCACGATGAAG<br>TGCGCGGTCTCGGGCTTGGTGACTGCGGGCGCTGTGAAGGACGCGCGATAGATGTTTTCGGCGCCGTTCGCGGGGATAGACGTCTTCCACG<br>TCCCCGCCTCAGGATAGTTGAACCAGAGAAACGACAGGCTGTCGCC
Gblock used for the KO of *mcpO*	TGCGCCCCTGAACGATGGCCCGACGTTCAAGGTTCAGCAAGATTTACCatgCCTTCTCTCGCCACAGTAGAGTTTGGGGGCGTGAAGATGGCC<br>GGTGCGTTCAAGCGGTTATCTCTGGCGGGGAAACTGATGGCGGCCGGGGGCGGGGCCCTTGGCGCGCTGTTGATCGCAGCCTCGCTGCTG<br>ATCGCCTGGAGCAGCAATGACGCTGTTCGAGACCTGGCCGAACGCTACGCCGCCAGCATGACCGGAGCCCTTTAGCTTTAGATGGAATCATC<br>TAAAGCGTTTGAAAGGGCTCTAAGTATCTGATTTTAGAGCCTGTTTATCTTGTTTAGATGGTTCCATCTAAACCAGACGGGCTCTAAGGCGCG<br>AACGCCGCCTCAGGCCGAGGCGGTTTCGATCTCGCTCATCACCGCCGCGCGCCAGCCTCTTTCGAGCGTCCAGGCGGCGATGGCCAGCCAG<br>AGCAGGCCCAGGAACGCGTGCCCGCGCTGCCAGTAG
Gblock used for the KO of *mcpP*	ACGTAGACACGCGTATGCGACAAAATGCGCGCGATCGCACTGAAGTCACGGACGGTTAATCCCTAGATCCAATGCTTGGCCGGAATCTCCA<br>GGGGCGGCCatgATCGAGTTCGACAGCATTGAGGCCCAGCGGCGCACGGGCGGGAAGCTGGTCTTGGCCTGCAATGCGGCGATGGTGGCG<br>TTGACGCCACTGGCCAGTCTTGTCGCGGGCAACGCGGTCGCGAAACTGGCCGGAATCGCCACGGTCCTGTCGGCTTCTGGCATCTGCGCTGA<br>ACCGCCTTGCGGCGGCGGGTCCAGAACAGCGAACGCGCTGGAGCGCTTTCCGATCCGTATGAAGCGGTGATCGAATGAAGAGCCGTCTACC<br>GTAAAGGTCAGCGAGCGGCGCAGCGCACAGCGGCCCTTTCGAGGCTCCAGTAGCGCCGGATGACCAGGGCGAGGGTCGCCAGCGTGAAG<br>CCGGCGGCGACGTAGCACGCTGGGCAATGGCCCGCGAACGCCCAGCT
Gblock used for the KO of *mcpQ*	AGTTCCACGCCGCCTTCGGCCGCTATTTCGAGATCGCCGCCGTTTACCGTCAGGTGGCGCTGGTGAAGACCTCCGAAATCCCGCGCTAGCGA<br>CGGTTCTCTAGAAATTCATTTAAATACAATATTTTAGGTATTTCGCCGACCATAGGTCTGCGACATCATGCCACCCATGCGAGAATGTCATGGT<br>TAAGGCCCGCTAGACTAGTTGGGCCGCATGCTCCGCCCATTCGGATCTGCGGAGAGGTCGCGATCACCGCCGCCAGCCATACCCTGGCGCA<br>GGAAGCCACACGACTGGCGCAGCGCATGGGCGAACTGCGGATCATGCGCGCCGGAATGCAGGCGGCGGCCTAGAGCGTTTTCCGATCTGT<br>TTGGATCGGAGAAACGCTCTAGGCCTTTGTATTAACGCATTTTCTGACGACGAACCGGATCCACTTCGTCGGAAAATGCTCTAGATCACACGC<br>CGCCGGCGGTCGAGGAACGGGCCGGTCGCGGGGTCTAGAG
Gblock used for the KO of *mcpR*	GACGTTAATGCTATGTTAGCCGTTTTGCTCGACCTCATTAACCTAATACGttgCGGGCGCGGCGTGCGAGCGGGGGGCTCGGGGATATGTGG<br>TTCAAAGAGATCGCCCGACGGCGTCGCGCCGAGACGCGCCGCCTGAAGGAACTGGAGAGCCTGGCCAAGGCCATGGAGCGGTCGCAGGCC<br>GTGCTGGAACTGCGGCCCAATGCGACGGTCGTCCGCGCCAACGGCGCGTTCCAGCGCCTGACCGGCTAGGGATGCTGTGTCATCCCGGAAA<br>GCGTGTAGCGCTTGTCCGGGACCCAGGGGCGGCAAGCGCGGCGCTCCCGCAGTCCCTGGGTCCCGGCTCTACCCCCGGCTTTCGCCGGGGT<br>TCGGCCGGGATGACACAGGATATTTCTGTCAGGGGTGGCGGAGGTGGCGAGCATGTGCGTCGATCCTGCGACTCACGAAACCCGAGACCCT<br>CCATGAAGTCCCTGTTCGCCGCCTCGGCTCTCGCCCTGCTGATCG
Gblock used for the KO of *mcpS*	ACCACGCCGACGACGCCGACGACATGTTCAGCCGCCTTATGGGTGACTTGGTCGAGCCGCGCCGAGAGTTCATCCAGGAGAACGCCCTCGA<br>CGCCGAGGTCGACGTCTAAAGCTCATCCTTGCAGCTAAGTGTTGTTGCAAGTGTTTGTTCCATAAGCGCCGGGCGCCCAATCCAGGGGCGGC<br>CCGGCGTTTTCTTTTCCAGAAACGCCCGTCTCGCCTTGGCCGCGGGCCCCAGTCGCTTCGCGTCGCCGCGGGAATGTTGAACGCTCGCGCAG<br>AGAAAGAGTACGCATTTGTTATCGTCCGCGTCCAACCCAATACGGGATGGCATGCTTCCGGGCGCCTTCCTGCGGAATTTTTGAACTAGTTTT<br>TCGCCCTGAAAGCGAATTCGTTCAGAACTCTGGCGCCGTCGTTGGATTCGATATGACATTTGTTGTCTTGCCAGCGTCATCATGTTTGTATAGC<br>GCATTGCCGGCTTGCTGTTTCAGTCGCTGGACACAACC

Table S2: Strains, primers and plasmids (4/4)

Target	Plasmid	Forward Primer (5'-3')	Reverse Primer (5'-3')
Primers used to control the deletion of *cheAI*		GCCCGAAAACCTGGATCT	CGAAGCTGCAGTACTCGTTG
Primers used to control the deletion of *mcpA*		TGTTCCGATCGGAAAGACGC	CCCGTAAGTCCTAATTCGCG
Primers used to overexpress *mcpA*	pMR10 (with HindIII and XbaI)	CCAAGCTTatgTTGGCGATCCGTGGG	GCTCTAGAttaGAATTCCTCCCAACC
Primers used to control the deletion of *mcpB*		CGAAGGCTTCAAGGTCGC	CCGCAATCTTACCGTGCG
Primers used to overexpress *mcpB*	pMR10 (with HindIII and XbaI)	CCCAAGCTTatgGGGACCGCCATGAAC	GCTCTAGAGCctaGAATTCTTCCCACTCTTCC
Primers used to control the deletion of *mcpC*		CCCTCTCTTTGCGGATGAGC	ACAAGGTCGGCAAGTTCCCG
Primers used to control the deletion of *mcpD*		ATGGCCTGGATCACCTCGGC	TCAGTGGTCGTCGCGTGAAGC
Primers used to control the deletion of *mcpE*		TCTACCGCGTTCGCTACAG	CCTGCCCAGAAATAGCTGTC
Primers used to control the deletion of *mcpF*		TTCGTGCACGACCAGTTCGC	GTCGCGCTTGCTCAAGTTCG
Primers used to control the deletion of *mcpG*		ATCGTCAAGCCTTTCAGCGC	CGGTGGGACAGATCAAGGC
Primers used to control the deletion of *mcpH*		AGAGGTTCTGGTTCCAGCGC	CGATACTCGTTCCAGGCCCG
Primers used to control the deletion of *mcpI*		ACATCGGGCTCTCCGAACGC	AATCTAGCGGCCTCGCTCCG
Primers used to control the deletion of *mcpJ*		GATAGCGCATCCAGTCAGC	GTTCGCGTTGCCTTCACCG
Primers used to control the deletion of *mcpK*		CTCATCCAGGTTCGTGAACCCGAGC	ATGGTCTGAGGACCCAGGCTCTCGC
Primers used to control the deletion of *mcpL*		GGCGATGATCTGAGTGTCGC	GCTTTCCGAGGAGCAGTTCG
Primers used to control the deletion of *mcpM*		TTCGCGCATCACTGAGATGC	AGACGGTGTGATCCTCACCCCGC
Primers used to control the deletion of *mcpN*		GGGTGACCACCTTGATAAGC	TTCTGAACCTGATCCCCACG
Primers used to control the deletion of *mcpO*		GTGATCACCGGCGACTTGC	GGCCTGTTGCTGGTCTTCG
Primers used to control the deletion of *mcpP*		GAGGGTCATCAAGCGAAGC	GGATCGTGTGTGAGACCAGC
Primers used to control the deletion of *mcpQ*		AGGCGATCCGCTACATGGAGATCGGC	ACAGCAGGGCCACAATCGCCATGC
Primers used to control the deletion of *mcpR*		GGATGGTCAAGCGCTACGGC	CCATGTGGATGTCGTGGCCG
Primers used to overexpress *mcpR*	pMR10 (with XhoI and BamHI)	CCCTCGAGttgCGGGCGCGGCGTGC	CGGGATCCCGGACAAGCGCTACACGCTT
Primers used to overexpress *mcpR* , *mcpRH99A* and *mcpRC75S* (for purification)	pET28a (with NdeI and BamHI)	GGAATTCcatatgttgCGGGCGCGGCG	CGggatccCGGACAAGCGCTACACGCTT
Primers used for the H99A mutation (with Q5 Site-Directed Mutagenesis Kit)		GCGCGCGCTGgctCGCGGCGAGC	CAGAATTCGCGGTACTCATCGCTG
Primers used for the H99A mutation (with Q5 Site-Directed Mutagenesis Kit)		ATTGTCGGGTctCCCCACAGCCTG	CTCCGGAGACGCGTAGCC
Primers used to control the deletion of *mcps*		ATCTCTACGCCGAGGAAGGC	GGCGAAAGAGCCGCAAAGC
Primers used to overexpress *sodB*	pMR10 (with XbaI and EcoRI)	GCTCTAGAatgCAACGCTTGCTGACCG	CGGAATTCctaGCGCGCGGCGGCG
Primers used to overexpress *katG*	pMR10 (with XbaI and XhoI)	GCTCTAGAgtgATGGAAACACCTGCGCG	CCCTCGAGctaAGCCGCCAGATCCAGGC
